# *Clostridioides difficile* infection (CDI) treatment outcomes and recurrence factor at a pediatric hospital

**DOI:** 10.1017/ash.2022.107

**Published:** 2022-05-16

**Authors:** Martin Tuan Tran, Jasjit Singh, Wendi Gornick, Beth Huff, Negar Ashouri

## Abstract

**Background:** CDI is the single most common cause of nosocomial diarrhea in both adults and children. Available data regarding treatment outcomes in hospitalized children remain limited. CDI recurrence in children has been reported in 20%–30% of cases. Consensus regarding the best testing method for CDI is lacking. The 2018 IDSA guideline recommends a multistep algorithm with detection of glutamate dehydrogenase antigen plus toxin, followed by detection of toxigenic *C. difficle* with nucleic acid amplification test (NAAT) if results are discordant. **Methods:** We included patients aged 1–26 years admitted from July 2020 through June 2021 with CDI symptoms and positive toxin or NAAT. Healthcare facility-onset CDI (HO-CDI) was defined as positive specimen collected >3 days after admission. Community-onset CDI (CO-CDI) was defined as positive specimen collected ≤3 days after admission. Community-onset healthcare facility-associated CDI (CO-HCFA-CDI) was defined as positive specimen from a patient who was discharged from the facility ≤4 weeks prior. Recurrence was defined as an episode of CDI occurring within 60 days after onset of a previous infection. **Results:** Mean age of the 63 patients meeting inclusion criteria was 11.2 years (range, 1–21 years). Most patients (n = 37; 58.7%) were male, tested negative for *C. difficile* toxins (n = 39; 61.9%), and had mild-to-moderate disease (n = 61; 96.8%). Patients with immunocompromising conditions were common, including malignancy (n = 38; 60.3%), inflammatory bowel disorder (n = 8; 12.7%), and history of solid organ transplant (n = 5; 7.9%). Previously healthy without chronic medical conditions were uncommon (n = 4; 6.3%). CO-CDI was most common (n = 26; 41.3%) followed by HO-CDI (n = 23; 36.5%). Also, 34 patients (53.9%) were exposed to antibiotics within the previous 30 days, 16 (47.0%) of whom received 2 or more antibiotics. Sulfamethoxazole–trimethoprim was the most prescribed agent (13; 38%), most (12; 92.3%) as prophylaxis for *Pneumocystis jirovecii* pneumonia. Furthermore, 42 patients (66.7%) were receiving gastric acid suppressant agents. Laxatives were given to 14 patients (22.2%) within 72 hours of testing, despite electronic reminders. Most were treated with oral vancomycin (n = 46; 73.0%). In addition, 5 patients (7.9%) did not receive CDI treatment at the discretion of the treating physician; all were toxin negative. CDI was cured in 58 patients (92.1%) with only 5 (7.9%) experiencing recurrence infection. Patients testing positive for *C. difficile* toxin were more likely to experience infection recurrence compared to those with a negative toxin screen: 4 of 24 (16.7%) versus 1 of 39 (2.6%) (*P* = .044). **Conclusions:** Most patients with CDI were treated with oral vancomycin at our institution. We observed significantly lower rate of recurrence than previously reported. Toxin-positive patients experienced higher recurrence rate. Prospective studies are needed to confirm our findings.

**Funding:** None

**Disclosures:** None

